# YOLO-PAM: Parasite-Attention-Based Model for Efficient Malaria Detection

**DOI:** 10.3390/jimaging9120266

**Published:** 2023-11-30

**Authors:** Luca Zedda, Andrea Loddo, Cecilia Di Ruberto

**Affiliations:** Department of Mathematics and Computer Science, University of Cagliari, Via Ospedale 72, 09124 Cagliari, Italy; dirubert@unica.it

**Keywords:** computer vision, deep learning, image processing, malaria parasite detection, early malaria diagnosis

## Abstract

Malaria is a potentially fatal infectious disease caused by the *Plasmodium* parasite. The mortality rate can be significantly reduced if the condition is diagnosed and treated early. However, in many underdeveloped countries, the detection of malaria parasites from blood smears is still performed manually by experienced hematologists. This process is time-consuming and error-prone. In recent years, deep-learning-based object-detection methods have shown promising results in automating this task, which is critical to ensure diagnosis and treatment in the shortest possible time. In this paper, we propose a novel Transformer- and attention-based object-detection architecture designed to detect malaria parasites with high efficiency and precision, focusing on detecting several parasite sizes. The proposed method was tested on two public datasets, namely MP-IDB and IML. The evaluation results demonstrated a mean average precision exceeding 83.6% on distinct *Plasmodium* species within MP-IDB and reaching nearly 60% on IML. These findings underscore the effectiveness of our proposed architecture in automating malaria parasite detection, offering a potential breakthrough in expediting diagnosis and treatment processes.

## 1. Introduction

Malaria, a widespread disease, is induced by the *Plasmodium* parasite and is transmitted to humans via bites from infected female Anopheles mosquitoes. In the year 2019, there were approximately 229 million reported cases of malaria globally, leading to 409,000 fatalities. Significantly, 94% of both malaria cases and associated deaths were concentrated in Africa, with children below the age of five identified as the most-susceptible demographic, constituting 67% of the total malaria-related deaths worldwide.

Although methods for the clinical diagnosis of malaria, such as molecular diagnostics with Real-Time Polymerase Chain Reaction (RT-PCR) [1], have been proposed in recent years, microscopy is the most-appropriate method for the detection of malaria in the field [2] and for blood diseases or infections in general. They are detected through the analysis of blood cells using peripheral blood slides under a light microscope. Thus, in addition to the diagnosis of malaria infection [3,4,5], some examples include the detection of leukemia [6,7,8,9] or the counting of blood cells [10,11,12,13,14].

Malaria, a disease caused by parasites belonging to the genus *Plasmodium*, manifests in humans through the invasion of Red Blood Cells (RBCs). Transmission occurs via the bites of infected female Anopheles mosquitoes, commonly known as “malaria vectors”. There are five main types of parasites responsible for human malaria: *P. falciparum* (*P.f.*), *P. vivax* (*P.v.*), *P. ovale* (*P.o.*), *P. malariae* (*P.m.*), and *P. knowlesi* (*P.k.*), with the first two posing the greatest threat [15,16].

The life stages of these parasites within the human host include the ring, trophozoite, schizont, and gametocyte phases. Human malaria, as defined by the World Health Organization (WHO), is considered a preventable and treatable condition if diagnosed promptly. Failure to address the disease promptly may lead to severe complications such as disseminated intravascular thrombosis, tissue necrosis, and splenic hypertrophy [16,17,18,19].

Nevertheless, the symptoms induced by malaria often closely resemble those associated with diseases such as viral hepatitis, dengue fever, and leptospirosis, thereby complicating the diagnostic process [2,20]. Several diagnostic methods have been developed to overcome this problem. Nevertheless, the currently available diagnostic tools often neglect or fail to distinguish between non-falciparum types [2]. In addition, several factors complicate the identification of these species. For example, parasitemia is typically very low in *P.v.*- and *P.m.*-infected individuals [21]; *P.v.* and *P.o.* are characterized by the slow development of some of their sporozoites (early stage of schizonts), forming hypnozoites, which are difficult to detect [22]. Infections with non-falciparum species are often asymptomatic, which makes their detection even more difficult because infected people do not seek treatment at a health facility due to the lack of symptoms. These reasons include the need to keep infectious diseases under control, especially in underdeveloped countries with no medical centers nearby or capable of handling many patients [16].

Manual microscopy of Peripheral Blood Smears (PBSs) has several advantages for malaria diagnosis, including low cost, portability, specificity, and sensitivity [23]. However, there are many problems associated with this method. Examples include technical skills in slide preparation; lysis of red blood cells and related changes in parasite morphology (leading to errors in species identification); quality and illumination of the microscope; the staining procedure; the competence and care of the microscopist; and finally, the level of parasitemia [2].

Moreover, the manual process is typically laborious and time-intensive, and incorrect diagnoses may result in unwarranted drug administration, with potential exposure to associated side effects or severe disease progression.

Further problems for this type of analysis are caused by the fact that, in many cases, only microscopy or rapid tests are available as diagnostic tools. Several pieces of research have shown consistent errors in *Plasmodium* species identification by microscopists, such as missed *P.o.* infections with low parasite densities, *P.f.*-infected specimens misidentified as *P.m.*, and *P.o.* slides misidentified as *P.v.*, which could lead to ineffective treatment administration and increase the risk of severe malaria. Furthermore, it is common to fail to distinguish early trophozoites of *P.v.* from those of *P.f.*, especially when parasitemia is low, as well as *P.m.* from other *Plasmodium* species using a microscopic method [24]. The similar morphologies of the malaria species can also lead to mixed infections, mostly misdiagnosed [2]. These events can also lead to a worsening of the clinical picture.

Accurate and timely malaria diagnosis is crucial for effective treatment and preventing severe complications. While traditional methods like microscopy remain the gold standard, recent developments in deep learning, specifically deep Convolutional Neural Networks (CNNs), have shown promising results in malaria cell image analysis.

Several studies have explored the application of deep CNNs in malaria diagnosis at the single-cell level, emphasizing the importance of accurately identifying whether a cell is infected with the malaria parasite [25,26,27,28,29,30].

Despite the advances produced by these methods, the use of datasets composed of images presenting monocentric cells represents an overly ideal scenario in which salient and highly discriminating features can be extracted from the images. Of course, this is valid under the assumption that pathologists take crops manually or that detection systems provide perfect crops. However, this assumption is not verified in real-world application scenarios because the systems are fully automated, and therefore, the crops cannot always be accurate or perfectly centered [31,32,33,34,35].

Other challenges exist, such as discriminating between different *Plasmodium* species and managing the complexities associated with low parasitemia levels and asymptomatic infections. Consequently, the exact localization of parasites within cells, obtained through precise bounding box detection, could offer valuable insights for in-depth studies and detailed diagnosis [5,36,37]. Therefore, integrating deep learning techniques with object detection capabilities becomes essential in this context. This integration allows accurate classification of infected cells and precise localization of parasites within these cells, providing comprehensive information for detailed analysis and diagnosis.

The challenges expressed motivated this work. Its objective was to devise a methodology named the Parasite Attention Module (PAM), which was seamlessly incorporated into the You Only Look Once (YOLO) architecture. This methodology was designed to automatically detect malaria parasites, addressing the limitations associated with the prevailing gold-standard microscopy technique. Specifically, the main contributions of this research are summarized as follows: (i) the development of a novel Transformer- and attention-based object-detection architecture based on the latest version of YOLO for malaria parasite detection; (ii) the investigation and extension of the proposal to the four different species for mixed or intra-species detection; (iii) the evaluation of two different datasets, including intra-dataset experimentations, based on the different species.

The rest of this article is organized as follows. First, the related work is presented in Section 2, and then, the materials and methods are described in Section 3. The proposed architecture is described in Section 4, while the experiments and results are presented in Section 5, along with a discussion of every investigation. Finally, the conclusions are drawn in Section 6.

## 2. Literature Review

In recent years, the field of computer vision has proposed various Computer-Aided Diagnosis (CAD) solutions aimed at automating the detection of malaria parasites. These endeavors seek to alleviate the challenges associated with manual analysis, offering a more-reliable and standardized interpretation of blood samples. This automation, in turn, can potentially mitigate diagnostic costs [37,38].

Before the emergence of deep learning techniques, malaria parasite detection in images relied on classical methods involving multiple steps: image preprocessing, object detection or segmentation, feature extraction, and classification. Techniques like mathematical morphology for preprocessing and segmentation [31,32], along with handcrafted features [33,34], have been used to train machine learning classifiers. The landscape of computer vision approaches for malaria parasite detection underwent a significant transformation with the introduction of AlexNet’s Convolutional Neural Network (CNN) [39], marking a paradigm shift.

Various deep learning approaches have been proposed as alternatives to classical methods for this task, as evidenced by numerous studies published in the last decade [5,25,30,36,37,40].

In the context of deep learning approaches, existing works on malaria can be divided into two categories. Works that perform classification on images containing single cells aim to identify the most-appropriate classifier to discriminate between parasitized and healthy cells by proposing custom CNN architectures or using off-the-shelf CNNs [25,26,27,28,29,30,41]. Additionally, Rajaraman et al. explored the performance of deep neural ensembles [30]. These methods typically use the NIH [29] dataset as a reference. More recently, Sengar et al.examined the use of vision Transformers on the same dataset [42].

On the other hand, works proposing full pipelines typically propose parasite detection from whole images and rely on several existing datasets, such as BBC041 [36], MP-IDB [43,44], IML [40], or M5 [37].

Arshad et al. proposed a dataset containing *P. vivax* malaria species in four life cycle stages. The authors presented a deep-learning-based life cycle stage classification, where the ResNet-50v2 network was selected for single-stage multi-class classification [40].

Sultani et al. collected a new malaria image dataset with multiple microscopes and magnifications using thin-blood smear slides. They obtained two variations of the dataset, one from Low-Cost Microscopes (LCMs) and another from High-Cost Microscopes (HCMs), aiming to replicate the challenges associated with real-world image acquisition in resource-limited environments. To address the malaria detection task, the authors used several object detectors. In addition, they also discussed the issue of microscope domain adaptation tasks and tested some off-the-shelf domain adaptation methods. The optimal performance emerged through the application of ranking combined with triplet loss, with the HCM serving as the source domain and the LCM as the target domain [37].

Since malaria parasites consistently target erythrocytes, automated malaria detection systems must analyze these cells to determine infection and classify the associated life stages. Existing literature only addresses the classification problem without considering the detection problem. Additionally, considerable emphasis has been placed on developing mobile devices to facilitate cost-effective and rapid malaria diagnosis, particularly in underdeveloped regions where access to more-expensive laboratory facilities is limited [34].

Regarding dataset utilization, some studies have employed the same datasets as utilized in this investigation. As of the current writing, a limited number of studies have used MP-IDB [5,44,45], whereas IML has only been utilized by its proposers [40]. Maity et al. implemented a semantic segmentation technique followed by the application of a Capsule Network (CapsNet) for the categorization of *P.f.* rings [5], whereas Rahman et al. conducted a comparative evaluation involving various off-the-shelf networks for binary classification purposes [45].

The principal distinctions between our study and the existing state-of-the-art methodologies stem from deploying a detector with a dual objective: identifying distinct types of malaria-infected RBCs and discriminating various life stages within a unified framework.

Compared to the works defined so far, this work aimed to provide a lightweight and effective method to detect malaria parasites of any species and life stage.

## 3. Materials and Methods

This section presents the materials and methods used in this study. Specifically, Section 3.1 gives an overview of the employed datasets. Then, Section 3.2 describes object detection with a specific focus on the YOLO family. Section 3.3 explains the modules composing the proposed architecture’s structure, and finally, Section 3.4 presents the metrics adopted to evaluate the experimental results.

### 3.1. Datasets

**MP-IDB** [4] comprises 210 images encompassing four distinct species of malaria parasites. The distribution is as follows: 104 images for *P. falciparum*, 37 for *P. malariae*, 29 for *P. ovale*, and 40 for *P. vivax*. Each parasite species exhibits four distinct life stages, namely ring, trophozoite, schizont, and gametocyte. Each image is accompanied by its associated ground truth, indicating the presence of one or more life stages. The entire dataset was captured at a resolution of 2592×1944 px with a color depth of 24 bit.

**IML** [40] contains images of blood samples taken with a camera mounted on an XSZ-107 series microscope at a 100× magnification. The dataset contains 345 images with an average of 111 blood cells. Only ***P. vivax*** is represented.

**Figure 1:**
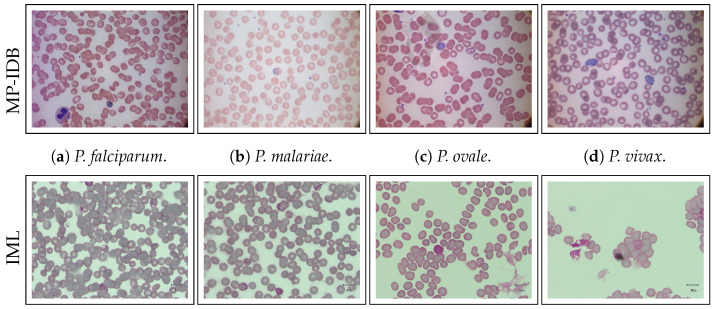
Comprehensive overview of the investigated datasets. The figure presents a detailed overview of the two datasets investigated in this study: MP-IDB and IML. MP-IDB encompasses four distinct malaria species—*P. falciparum*, *P. malariae*, *P. ovale*, and *P. vivax*. In contrast, the IML dataset exclusively consists of samples related to *P. vivax*. Notably, the MP-IDB dataset demonstrates intra-species variations, while the datasets differ significantly from each other.

Sample images from both datasets are presented in Figure 1.

### 3.2. Object Detectors

Modern detectors are based on deep learning methods and are divided into two categories: one-stage and two-stage. Two-stage architectures, such as Faster R-CNN [46], first extract Regions Of Interest (ROIs) and, then, perform classification and bounding box regression in a coarse-to-fine process. In contrast, one-stage detectors, including the SSD [47], FPN [48], and YOLO family [49,50,51,52], produce bounding boxes and classes directly from predicted feature maps with predefined anchors.

One-stage detectors are faster and more compact, making them more suitable for time-critical applications and computationally constrained edge devices [53,54].

Recently, the success of Transformers in image recognition has led to the development of several end-to-end Detection Transformers (DETRs). Despite their high recognition accuracy, DETRs are hampered by their complex architectures and slow convergence problems [53].

To overcome these limitations, in this paper, we propose a modified version of the one-stage detector YOLOv8 to be efficient and accurate, especially on small parasite objects.

#### YOLO

Instead of the traditional two-step approach based on a region-selection method, the YOLO family of detectors uses an end-to-end differentiable network that integrates bounding box estimation and object identification. YOLO divides the input image into S×S constant-size grids, and a CNN predicts the bounding boxes and classes for each grid. If the confidence of a bounding box is above a certain threshold, it is selected to locate the object in the image. The CNN performs only one pass to make predictions and, after non-maximum suppression, produces known objects and their bounding boxes, ensuring that each object is detected only once.

YOLOv8 is a family of architectures and models for object detection pre-trained on the Common Object in Context (COCO) dataset [55].

This family comprises five distinct models that share a common architecture, but diverge in breadth, depth, and the number of trainable parameters. The models denoted as *YOLOv8n* (nano), *YOLOv8s* (small), *YOLOv8m* (medium), *YOLOv8l* (large), and *YOLOv8x* (extra-large), are each pre-trained on images with resolutions of either 640×640 or 1280×1280 px. Notably, in terms of trainable parameters, YOLOv8n encompasses 3.2 million, YOLOv8s 11.2 million, YOLOv8m 25.9 million, YOLOv8l 43.7 million, and YOLOv8x 68.2 million.

The YOLOv8 architecture is composed of three integral components, similar to other single-stage object detectors, namely the backbone, neck, and prediction head.

The backbone is a pre-trained network specialized in extracting features from the input image. This process involves reducing the spatial resolution of the image while concurrently increasing the resolution of the extracted features.

The neck component combines the extracted features and generates three distinct scales of feature maps, commonly referred to as feature pyramids. This design enhances the model’s ability to generalize effectively to objects of varying sizes and scales.

Subsequently, the prediction head employs anchor boxes on the feature maps, facilitating the detection of objects based on the previously generated feature maps.

Similarly to YOLOv5, the YOLOv8 architecture uses the CSPDarknet53 architecture with a Spatial Pyramid Pooling (SPP) layer [56] as the backbone, uses the Path Aggregation Network (PANet) [57] as the neck, and the YOLO detection head [49].

Despite the significant improvement in detection speed, it is a well-known fact that YOLO architectures struggle to detect small objects, compared to two-stage detectors [49,54]. This particular problem was addressed as one of the objectives of this paper. In fact, the considered scenario included cases where small parasites appear. The smallest ones, i.e., the smallest rings, are sometimes not large enough to be considered by a generic detector.

### 3.3. Core Modules and Mechanisms

**Figure 2:**
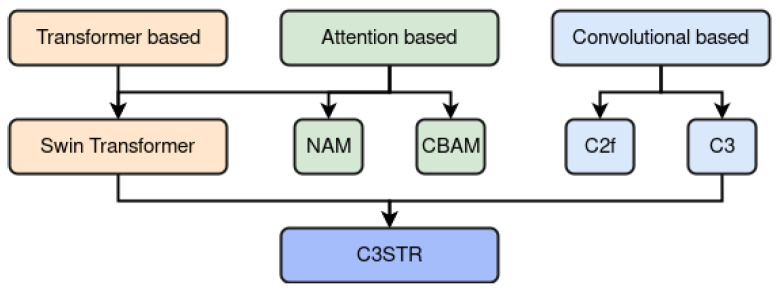
Overview of the modules and mechanisms’ hierarchy proposed in this study to enhance the performance of YOLOv8. Here, NAM stands for Normalized Attention Module, while CBAM refers to Convolutional Block Attention Module. Further, C2f is a fast implementation of the Cross-Stage Partial (CSP) Bottleneck with 2 convolutions, while C3 indicates a CSP Bottleneck with 3 convolutions. Finally, C3STR refers to the integration of the Swin Transformer Block in place of the C3 module’s Bottleneck.

In this section, we review the several key components and state-of-the-art mechanisms adopted in order to enhance the existing baseline architecture. To better visualize the relationships and hierarchy discernible among these components, we provide a schematic representation in Figure 2.

#### 3.3.1. Attention Mechanisms

The concept of attention, a vital cognitive function in human perception, involves selectively focusing on the salient parts of a scene, enabling efficient processing of visual information [58]. This ability allows humans to filter relevant information with limited computational resources, enhancing both efficiency and accuracy in perception [59].

In recent years, attention mechanisms have found applications in various computer science domains, including natural language processing and Computer Vision (CV) [59]. In these contexts, attention acts as a technique to emphasize specific parts of input data when generating output, essentially assigning importance weights to different input features.

In the realm of object detection, attention mechanisms guide the model’s focus toward image regions likely to contain relevant objects. This selective attention significantly enhances object detection accuracy by reducing irrelevant information processed by the model. An approach in computer vision involves utilizing CNNs with attention modules [60]. These modules consist of learnable weights that prioritize different regions of the input image. Through training, the model adapts these weights, learning to concentrate on crucial image regions for a given task.

With regard to CV tasks, attention mechanisms can be broadly classified into channel, spatial, temporal, branch, channel and spatial, and spatial and temporal attention [61]. For the purpose of this work, we provide a concise definition of the channel and spatial attention mechanisms. On the one hand, channel attention dynamically modifies the importance of each channel, resembling the selection of specific objects and, thus, determining what deserves attention. This adjustment follows the concept that, in deep neural networks, distinct channels within various feature maps typically signify separate objects [61]. Hu et al. [62] introduced channel attention and proposed SENet for this task.

On the other hand, spatial attention involves creating an attention mask that spans various spatial domains within an image. This mask is generated to highlight essential regions. These highlighted regions are either selected directly as important spatial areas based on the generated attention mask or the attention mechanism predicts the most-relevant spatial positions directly [61]. This process enables deep learning models to focus on specific parts of an image, improving their ability to recognize objects and understand the context within complex visual data.

#### 3.3.2. Convolutional Block Attention Module (CBAM)

To enhance informative channels and important regions of CNNs, Woo et al. [58] proposed the Convolutional Block Attention Module (CBAM). It sequentially stacks channel and spatial attention modules, which decouple the channel and spatial attention maps for computational efficiency. Additionally, CBAM leverages spatial global information by introducing global pooling.

CBAM has two sequential sub-modules, channel and spatial. Given an input feature map $X\in R^{C\times H\times W}$, CBAM sequentially infers a 1D channel attention vector $s_{c}\in R^{C}$ and a 2D spatial attention map $s_{s}\in R^{H\times W}$.

The channel attention module learns to weigh the importance of different channels in a feature map based on their relevance. In contrast, the spatial attention module understands to selectively emphasize important spatial regions of the feature map. This combination permits the highlighting of proper channels and enhancing informative local regions.

CBAM can also be represented in the notation expressed by Equation (1):

(1) $$W_{c}=\sigma \left(\mathrm{MLP}\left(A_{\mathrm{pool}}\left(X\right)\right)\right),$$

where $A_{\mathrm{pool}}$ denotes a global average pooling operation that aggregates the spatial dimensions of the feature map, MLP denotes a two-layer feedforward network with ReLU activations, and σ represents the sigmoid activation function.

The spatial attention module operates on the feature map *X* and the channel attention weights $W_{c}$. It first computes a set of spatial attention weights $W_{s}$ by passing the feature map through a convolutional layer with a sigmoid activation function, as shown in Equation (2):

(2) $$W_{s}=\sigma \left({\mathrm{Conv}}_{7x7}\left(A_{\mathrm{pool}}\left(X\right)⊗W_{c}\right)\right),$$

where ⊗ denotes elementwise multiplication, Conv denotes a convolutional layer, and $A_{\mathrm{pool}}$ is as defined above. The spatial attention weights are then used to modulate the feature map as follows (defined in Equation (3)):

(3) $$Y=W_{s}⊗X,$$

where *Y* denotes the output feature map.

#### 3.3.3. Normalized Attention Module

The Normalized Attention Module (NAM), proposed for neural networks, represents a lightweight and efficient attention mechanism. This module combines the channel and spatial attention mechanisms into a unified module, utilizing the batch normalization scaling factor to measure the importance of both channels and pixel regions [63].

This module can be described by the following notation:

(4) $$M_{c}=\mathrm{sigmoid}\left(W_{\gamma }\left(\mathrm{BN}(F_{1})\right)\right)$$

(5) $$M_{s}=\mathrm{sigmoid}\left(W_{\lambda }\left({\mathrm{BN}}_{s}\left(F_{2}\right)\right)\right)$$

where $W_{\gamma }$ and $W_{\lambda }$ are scaling factors, BN is a batch normalization module, and $F_{1}$, $F_{2}$ are the input feature maps.

#### 3.3.4. Swin Transformer

This is one of the most-recent Vision Transformer architectures, which has shown impressive results in object detection tasks [64] and is the current state-of-the-art on the COCO test–dev dataset. The core idea of the Swin Transformer is to use hierarchical partitioning of image feature maps, allowing for efficient computation and scalability to larger input resolutions. In particular, the Swin Transformer Block replaces the standard self-attention mechanism with a shifted window-based self-attention. This allows the attention mechanism to be computed more efficiently, as the computational complexity is reduced from quadratic to linear with respect to the input resolution. Additionally, the Swin Transformer Block introduces a hierarchical structure, which allows for multi-scale feature representation, which is critical for capturing information at different levels of abstraction in an image.

#### 3.3.5. C2f and C3 Modules

The C2f and C3 modules are core modules of the YOLO architecture. On the one hand, the C3 module belongs to the YOLOv5 architecture and is one of its core architectural Blocks. It basically indicates a CSP Bottleneck with three convolutions. On the other hand, the C2f module belongs to the YOLOv8 architecture and serves as an architectural upgrade, replacing the C3 module. The C2f module is faster and lighter, which is achieved by using a lower number of convolutional filters. It is a CSP Bottleneck with two convolutions.

#### 3.3.6. C2f

The C2f module consists of a 1×1 convolution layer called cv1, which reduces the input channels to twice the hidden channels, and a 1×1 convolution layer called cv2, which reduces the input channels to the desired output channels. It also includes a sequence of Bottleneck Blocks for further processing. The forward pass involves applying cv1 to the input tensor *x*, splitting the output into two parts, processing them through the Bottleneck Blocks, and finally, concatenating the outputs and passing them through cv2 to obtain the final output.

#### 3.3.7. C3

The C3 module is similar to C2f, but with an additional 1 × 1 convolution layer called cv2. It includes a 1×1 convolution layer cv1, another cv2, and a 1×1 convolution layer called cv3 for reducing the concatenated input channels to the desired output channels. The forward pass involves applying cv1 and cv2 to the input tensor separately, concatenating the outputs, processing them through the Bottleneck Blocks, and passing the result through cv3 to obtain the final output.

#### 3.3.8. C3 Swin Transformer Block

This is a C3 module with a Swin Transformer Block in place of the Bottleneck component. Some works, such as [65], have added the Swin Transformer Block to the YOLOv5 architecture, while we included the C3 Swin Transformer Block (C3STR) module within our architectures.

### 3.4. Metrics

The assessment of object-detection methods often involves the use of the mean average precision metric and its variations, as described in COCO [55]. Precision is calculated based on the concept of the Intersection over Union (IoU), which measures the accuracy of detection by comparing the overlapping area between the predicted bounding box and the actual object to the total combined area.

To determine the accuracy of detection results, a specific threshold is set for the IoU. If the IoU exceeds this threshold, the detection is considered accurate and classified as a True Positive (TP). On the other hand, if the IoU falls below the threshold, the detection is labeled as a False Positive (FP). Additionally, if the model fails to detect an object that exists in the ground truth, it is referred to as a False Negative (FN).

Experimental evaluations were conducted using five variations of the mean average precision metric:

- **AP** was evaluated with 10 different IoUs varying in a range of 50% to 95% with steps of 5%;
- **AP**${}_{50}$ was evaluated with a single value o the IoU corresponding to 50%;
- **AP**${}_{s}$ is the AP determined for small objects (with area $<32^{2}$ px);
- **AP**${}_{m}$ is the AP determined for medium objects (with $32^{2}<$ area $<96^{2}$ px);
- **AP**${}_{L}$ is the AP determined for large objects (with area $>96^{2}$ px).

## 4. The Proposed Network: YOLO-PAM

In addition to proposing an efficient and precise malaria-parasite-detection system, this study aimed to overcome the limitations of existing state-of-the-art methods. Our objective was threefold: First was to achieve the speed and compactness typical of one-stage detectors while maintaining high accuracy without the need for a secondary stage, i.e., the classification stage. Second, we integrated Transformer models, steering clear of an end-to-end DETR framework to avoid excessive complexity and sluggish convergence. Last, we tackled the detection of the parasites of varying the sizes, from small to large, within a unified system, eliminating the need for an additional specialized subsystem.

Our methodology primarily focused on enhancing the efficiency and accuracy of the medium-sized one-stage detector, YOLOv5m6. Selected for its characteristics, this model comprised 35.7 million trainable parameters and was pre-trained on images sized at 1280×1280 pixels. Modifying its final layer equipped it to effectively detect all four phases of the malaria life cycle. This model balances network depth and parameter count, making it highly suitable for low-end computational resources and mobile devices [66].

**Figure 3:**
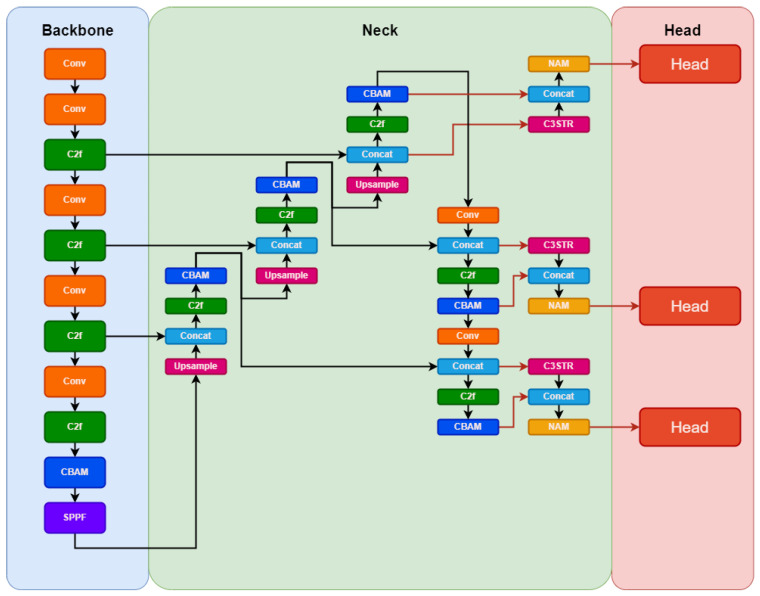
The proposed YOLO-PAM architecture.

Our proposed YOLO-PAM model aimed for a lighter approach compared to YOLOv5m6. To achieve this, we adopted the same fundamental concepts of YOLOv5m6 for the YOLOv8m architecture. We also reduced the model’s width to 3/4 of its original, resulting in fewer filters used and enabling faster training and inference. The architectural proposal is illustrated in Figure 3.

A key contribution involved strategically integrating multiple CBAM attention modules within vital components of the baseline architecture, such as the backbone and neck, influencing the prediction heads. This enhancement builds upon prior research [58], demonstrating the efficacy of these modules in improving classification and detection tasks. We made specific modifications to the original YOLOv8 architecture:

1. We excluded prediction heads designed for large objects, retaining those tailored for medium-sized ones. This decision aimed to prevent unnecessary computational overhead associated with handling excessively large objects and directed the architectural focus towards the precise dimensions of the objects in the images, specifically those of small and medium size in terms of pixel count.
2. Moreover, an additional head was incorporated to use features from the lower layers of the model’s backbone. These layers offer less-refined features, but possess higher resolution, a critical aspect for the precise detection of smaller objects, for example. Leveraging lower backbone layers allows the extraction of high-resolution features, essential for discriminating objects occupying minimal pixels. Higher layers excel in discerning medium-sized objects, but might lose the details of smaller objects due to the reduced feature map resolution caused by the convolution.
3. A further contribution entailed the integration of features extracted from the C3STR layers with those acquired from the C3 layers immediately followed by the CBAM layers. Subsequently, a NAM module was applied to introduce further attention to the resulting feature maps. This procedure endowed the prediction heads with the utmost refined features the model could generate, a detail highlighted by the orange arrows in the schematic representation illustrated in Figure 3.

In contrast to alternative strategies that integrate Transformers and attention mechanisms by substituting the last C3 layer with Transformer Blocks [65,67], our approach diverged significantly. Our objective was to preserve the distinctive locally specialized features intrinsic to CNNs while incorporating global features extracted through vision Transformers. This approach involved merging these two feature types within the model heads, enabling the retention of the nuanced advantages associated with global and local features. In summary, our strategy involved integrating three different attention mechanisms—CBAM, NAM, and C3STR—aiming to leverage their respective advantages.

## 5. Experimental Results

This section delineates the conducted experimental evaluation, starting with a comprehensive overview of the experimental setup detailed in Section 5.1. Additionally, it provides details regarding dataset splits and the implemented data augmentation, aiming to offer a comprehensive understanding of the experimental design. Subsequent sections are dedicated to specific aspects of the evaluation: Section 5.2 delves into the ablation study, while Section 5.3 and Section 5.4, respectively, present the results on MP-IDB and IML. Furthermore, Section 5.5 furnishes an overview of the qualitative results, and lastly, Section 5.6 undertakes a comparative analysis between our proposed architecture and the state-of-the-art methods.

### 5.1. Experimental Setup

The experiments were performed on a workstation with the following hardware specifications: an *Intel(R) Core*(TM) i9-8950HK @ 2.90 GHz CPU, 32 GB RAM, and an *NVIDIA GTX1050 Ti* GPU with 4 GB memory. We used the PyTorch implementation of YOLOv8 (available at: https://github.com/ultralytics/ultralytics (accessed on 28 November 2023)), developed by the Ultralytics LLC and the YOLOAir’s implementation of C3STR (available at: https://github.com/iscyy/yoloair (accessed on 28 November 2023)). The backbones used were ResNet-50 pretrained on ImageNet with the FPN and Darknet53 for YOLO. All YOLO-based networks were initialized using pre-trained weights on the COCO2017 dataset [55]. Adam served as the optimizer, configured with a learning rate of 0.001 and a momentum of 0.9. Each model underwent training for 100 epochs, employing a batch size of 4.

**Datasets’ split:** For MP-IDB, each parasite class was allocated a split of 60% for training, 20% for validation, and 20% for testing. The original splits proposed by the authors were retained for IML [40]. Detailed information regarding the dimensions of the parasites can be found in Table 1.
**Data augmentation:** We generated 35 distinct augmented samples from each original sample for every species. This augmentation strategy aimed to enhance the diversity of the training data, address data imbalance issues, bolster the models against potential object rotations, and enable targeted generalization capabilities. We chose a milder augmentation approach due to the vulnerability of certain parasites. Specific augmentation techniques, such as shearing, were observed to have the potential to adversely affect parasites, with notable implications for those of smaller dimensions [44]. Table 2 shows the applied augmentations.

### 5.2. Ablation Study

Table 3 presents the results of the ablation study conducted on the *P.f.* split of the MP-IDB dataset, specifically chosen due to its diverse representation of different life stages and the presence of parasites of varying sizes, ranging from small to large. The objective of these experiments was to systematically evaluate the impact of various modifications on the detection performance. Four different configurations were tested: The baseline method (YOLOv8m) achieved an AP of 78.9%. When incorporating CBAM alone, the performance improved marginally to 79.6%. By focusing solely on the modifications in the backbone architecture (C3), the AP score increased to 81.2%. The most-substantial improvement was observed when both the CBAM and C3 modifications were integrated (CBAM + C3), resulting in an AP score of 83.6%. This table provides a detailed insight into the effectiveness of each modification. It underscores the significance of the combined enhancements, demonstrating their positive impact on the accuracy of malaria parasite detection.

### 5.3. Experimental Results on MP-IDB

Table 4 presents a detailed quantitative assessment of malaria parasite detection performance across four species (*P.f.*, *P.m.*, *P.o.*, and *P.v.*) within MP-IDB. The evaluation employed multiple detection methods: Faster R-CNN, RetinaNet, FCOS, YOLOv8m, and the proposed YOLO-PAM.

Across the *P.f.* class, YOLO-PAM showcased remarkable performance, achieving an AP of 83.6%, outperforming other methods, including YOLOv8m (78.9%), and demonstrating significant improvements. It also surpassed Zedda et al.’s [44] method by a consistent 3.7% in the AP. In the *P.m.* category, YOLO-PAM excelled once again, achieving a striking AP of 93.6%, surpassing the baseline YOLOv8m’s AP of 78.8%. Similarly, in the *P.o.* class, YOLO-PAM achieved an outstanding AP of 94.4%, outclassing YOLOv8m’s AP of 89.7%. In the *P.v.* category, YOLO-PAM attained an AP of 87.2%, demonstrating superior performance compared to YOLOv8m’s AP of 85.9%.

Notably, YOLO-PAM consistently outperformed other methods across all parasite species, as evidenced by the bolded entries in the table. These numerical results underscore the effectiveness of the proposed YOLO-PAM in malaria parasite detection, emphasizing its accuracy and robustness in identifying parasites of varying sizes within different species.

### 5.4. Experimental Results on IML

Table 5 provides a detailed analysis of the malaria parasite detection performance across multiple methods within the IML dataset [40].

In the context of the overall AP, YOLO-PAM emerged as the most-effective method, achieving a remarkable AP of 59.9%, outperforming other methods such as the FRCNN (27.9%), RetinaNet (24.2%), FCOS (7.2%), and even the baseline, YOLOv8m (56.2%). YOLO-PAM’s superior performance is further highlighted by its excellent AP${}_{50}$ score of 91.8%, indicating its ability to accurately detect parasites with a high IoU threshold.

Moreover, regarding specific AP scores, YOLO-PAM excelled across different object scales. It achieved the highest AP${}_{m}$ (medium-sized objects) at 60.0%, demonstrating its precision in detecting parasites of medium sizes. Additionally, YOLO-PAM achieved a substantial AP${}_{L}$ (large-sized objects) score of 65.0%, underscoring its capability in accurately identifying larger parasites. These results emphasize YOLO-PAM’s versatility and accuracy across various object sizes, making it a robust and reliable choice for malaria parasite detection tasks within the IML dataset. It is important to note that IML does not provide any small parasites.

### 5.5. Qualitative Analysis

**Figure 4:**
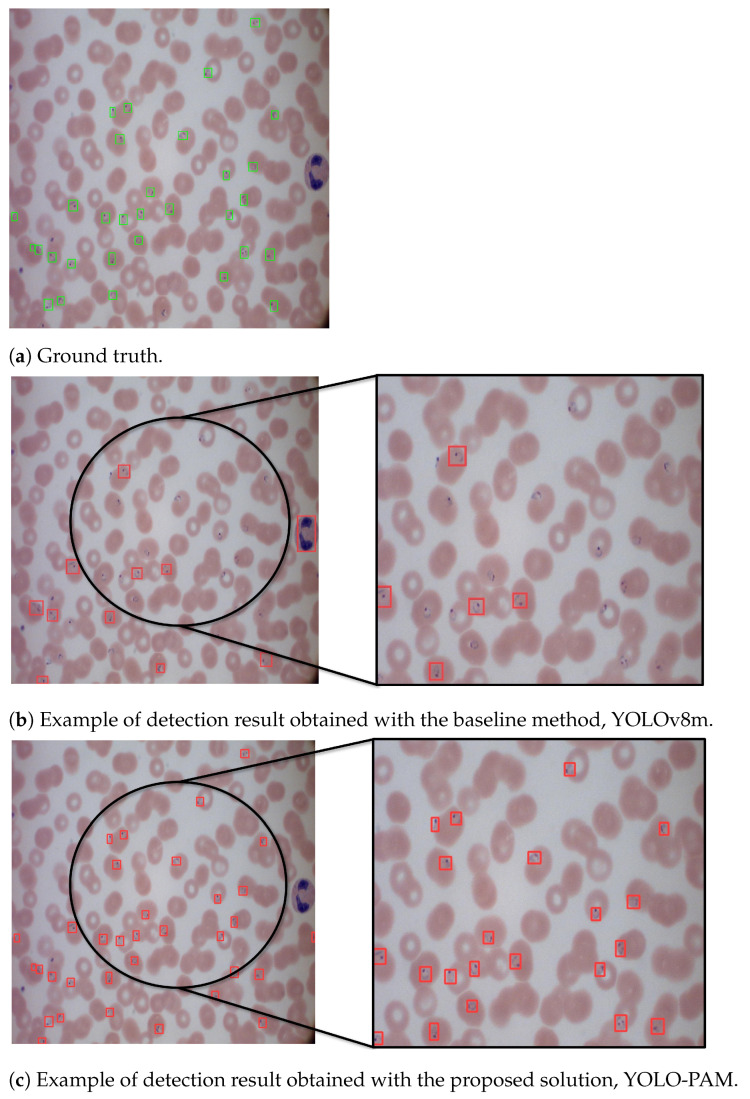
The middle section shows detection outcomes obtained with the baseline method, YOLOv8m, on a sample image taken from the *P.f.* split of MP-IDB. A closer look (at the right) reveals missing parasites in the detection, along with the misclassification of a white blood cell as a parasite. In contrast, the lower section presents results obtained with the proposed method, YOLO-PAM. Here, all the parasites are accurately detected, and the white blood cell is not flagged as a parasite. This comparison underscores the enhanced precision and accuracy achieved by YOLO-PAM.

Figure 4 shows the predicted bounding boxes generated by the proposed architecture. As can be seen, it demonstrated a high degree of agreement with the ground truth, showing outstanding improvements over the baseline results obtained with YOLOv8. Moreover, YOLO-PAM outperformed the detectors adopted for comparison, as shown by the numerical data presented in Table 4 and Table 5.

### 5.6. System Comparison

In this section, we compare YOLO-PAM with some works present in the literature that employed the same datasets as the object of this study.

Regarding the MP-IDB dataset, Rahman et al. conducted binary classification on individual cells segmented from MP-IDB using the watershed transform. Diverging from their approach, we focused exclusively on parasite detection, omitting healthy RBCs. Thus, a direct comparison was precluded. Nevertheless, the authors achieved an 85.18% binary classification accuracy using a fine-tuned VGG-19 specialized in discriminating healthy single RBCs from infected counterparts [45].

Maity et al. built a comprehensive system capable of segmenting infected RBCs using a multilayer feedforward Artificial Neural Network applied to full-sized images. They subsequently employed a CapsNet for the classification of the obtained crops. Their reported correct classification of 885 *P. falciparum* rings out of 927 yielded a classification accuracy of 95.46%. However, the authors did not extend the classification to gametocytes, trophozoites, or schizonts [5].

Zedda et al. used a modified version of YOLOv5 for parasite detection, reporting an 84.6% mean average precision on MP-IDB [44].

Concerning the IML dataset, Arshad et al. implemented a framework involving a segmentation step followed by multi-stage classification using off-the-shelf CNNs. Two segmentation methodologies were tested, yielding 89.33% precision with the morphological approach and 82.42% with the U-Net method [40].

The proposed YOLO-PAM approach offers several advantages compared to existing state-of-the-art methods. First, in the critical scenario of malaria parasite detection, where swift and accurate identification is paramount for timely diagnosis, YOLO’s efficiency in providing real-time results stands as a significant advantage. Furthermore, YOLO-PAM’s reduced parameter count, compared to other architectures, strikes a balance between accuracy and speed, which is particularly beneficial in resource-constrained settings where prompt and precise detections are imperative for timely intervention. Table 6 compares parameter counts and inference times between YOLO-PAM and other architectures, demonstrating improved results with lower parameters and comparable inference times to the reference baseline.

Second, YOLO-PAM provides a unified framework for end-to-end detection, allowing simultaneous predictions of bounding boxes and class probabilities for multiple parasite types and stages within an image.

Third, the unified architecture of YOLO-PAM, considering the entire image in a single forward pass, streamlines the detection process and enhances the model’s ability to capture spatial dependencies effectively.

By leveraging these advantages, this work aimed to contribute significantly to the field of malaria diagnosis by providing a robust and efficient solution for automated parasite detection.

## 6. Conclusions

In summary, this study’s experimental results and analysis demonstrated the effectiveness and superiority of the proposed malaria-parasite-detection method, YOLO-PAM, across multiple datasets and parasite species. The ablation study systematically assessed the impact of various modifications on the detection performance. Notably, integrating both CBAM and C3STR modifications significantly enhanced the accuracy, highlighting the importance of these combined enhancements.

When evaluated on MP-IDB, YOLO-PAM consistently outperformed existing methods across all four parasite species. Notably, within the *P.f.* class, YOLO-PAM achieved a remarkable Average Precision (AP) of 83.6%, surpassing both the baseline YOLOv8m and the previously established state-of-the-art detection method [44]. Similarly, in the *P.m.* and *P.o.* categories, YOLO-PAM exhibited high performance, demonstrating its precision in detecting parasites of varying sizes within these species.

Furthermore, the evaluation of the IML dataset reinforced YOLO-PAM’s superiority. With an overall AP of 59.9%, it outperformed the FRCNN, RetinaNet, FCOS, and the baseline, YOLOv8m, demonstrating its accuracy in detecting malaria parasites even in challenging scenarios.

YOLO-PAM exhibited precision for small objects, as observed in the *P.f.* class, and for medium- and large-sized parasites, underscoring its versatility across different object scales.

In conclusion, YOLO-PAM presents a robust and reliable solution for malaria parasite detection, addressing the limitations of existing methods and demonstrating superior performance across diverse datasets and parasite species. Its accuracy, versatility, and reliability make it a valuable contribution to malaria research and healthcare, promising significant advancements in malaria diagnosis and ultimately contributing to the global efforts to combat this infectious disease.

Several potential directions for future research were outlined. The primary goal was to enhance the current approach to accurately detect all malaria parasite species simultaneously. Additionally, building upon the encouraging results within the intra-dataset context, the approach will be tailored to a cross-dataset framework to enhance its resilience to potential environmental variations between the source and target data. Finally, a long-term objective is to expand the approach to encompass a multi-magnification image representation of the same blood smear, enabling more-precise detection of malaria parasites across varying magnifications.

## Figures and Tables

**Table 1:** Distribution of the parasites of both datasets based on their size, measured in pixels. S, M, and L indicate Small, Medium, and Large parasites.

		Parasites								
Dataset	Species	Train Set			Val Set			Test Set		
		S	M	L	S	M	L	S	M	L
MP-IDB	*P. falciparum*	370	408	0	123	136	0	123	136	1
	*P. malariae*	1	25	0	1	8	0	0	8	0
	*P. ovale*	0	20	0	0	6	0	0	7	0
	*P. vivax*	2	29	2	1	10	1	3	10	5
IML	*P. Vivax*	6	128	249	1	9	49	3	16	89

**Table 2:** Augmentation parameters and their associated probabilities. The table delineates the parameters employed for data augmentation, including rotation range iterations, Gaussian noise variance range, and Hue, Saturation, Value (HSV) shift limits, along with their respective probabilities of implementation.

Augmentation	Parameters	Probability
Rotation	range iterations: [0, 3]	1
Gaussian Noise	variance range: [50, 100]	0.3
HSV-Hue	shift limit: 20	0.3
HSV-Saturation	shift limit: 30	0.3
HSV-Value	shift limit: 20	0.3

**Table 3:** Ablation study conducted on the *P.f.* split of MP-IDB, chosen for its representative selection of various life stages and the presence of small, medium, and large parasites. This subset enables comprehensive assessment and validation of the proposed modifications and enhancements.

Method	AP (%)
Baseline (YOLOv8m)	78.9
Baseline + CBAM only	79.6
Baseline + C3STR only	81.2
Baseline + CBAM + C3STR (Our)	83.6

**Table 4:** Quantitative evaluation results obtained on the four parasite classes in MP-IDB [4]. The reported performance metrics include the average precision at different Intersection over Union thresholds and scales. The best results are emphasized in bold.

Class	Method	AP (%)	AP${}_{50}$	AP${}_{s}$	AP${}_{m}$	AP${}_{L}$
*P.f.*	FRCNN	39.2	80.6	33.7	44.3	0.0
	RetinaNet	34.0	78.5	23.9	42.6	0.0
	FCOS	10.1	39.9	5.6	14.5	0.0
	Zedda et al. [44]	-	95.2	-	-	-
	YOLOv8m	78.9	98.3	70.0	77.0	90.0
	YOLO-PAM	**83.6**	**98.9**	**76.0**	**80.0**	**99.9**
*P.m.*	FRCNN	75.1	98.4	-	75.1	-
	RetinaNet	76.0	95.0	-	76.2	-
	FCOS	4.7	21.2	-	8.8	-
	YOLOv8m	78.8	97.2	-	74.0	-
	YOLO-PAM	**93.6**	**98.5**	-	**84.2**	-
*P.o.*	FRCNN	71.0	89.1	-	71.0	-
	RetinaNet	74.3	91.5	-	74.3	-
	FCOS	44.2	81.8	-	45.1	-
	YOLOv8m	89.7	**99.5**	-	83.0	-
	YOLO-PAM	**94.4**	**99.5**	-	**85.0**	-
*P.v.*	FRCNN	60.3	87.7	20.2	61.5	85.0
	RetinaNet	62.8	85.5	10.1	65.7	84.1
	FCOS	53.0	81.0	5.1	53.8	83.1
	YOLOv8m	85.9	93.7	**19.0**	83.9	**96.0**
	YOLO-PAM	**87.2**	**94.2**	**19.0**	**86.0**	94.0

**Table 5:** Quantitative evaluation results obtained on the IML dataset [40]. The reported performance metrics include the mean average precision at different Intersection over Union thresholds and scales. The best results are emphasized in bold.

Method	AP (%)	AP${}_{50}$	AP${}_{s}$	AP${}_{m}$	AP${}_{L}$
FRCNN	27.9	73.1	-	31.9	0.0
RetinaNet	24.2	71.2	-	30.7	0.0
FCOS	7.2	36.2	-	10.5	0.0
YOLOv8m	56.2	89.2	-	55.5	64.0
YOLO-PAM	**59.9**	**91.8**	-	**60.0**	**65.0**

**Table 6:** Indication of the number of parameters (in millions) of every architecture used and inference time (in seconds). YOLO-PAM offer improved results with lower parameters and almost the same inference time as the baseline.

Models	Parameters (M)	Inf. Time (s)
YOLOv8 (baseline)	43.2	0.0131
FRCNN	41.2	0.101
RetinaNet	34.1	0.102
FCOS	32.3	0.286
YOLO-PAM	29.8 (−13.4)	0.0165

## Data Availability

All the material used and developed for this work is available at the following GitHub repository https://github.com/snarci/YOLO-SPAM (accessed on 28 November 2023).

## Abbreviations

The following abbreviations are used in this manuscript:

*P.*	*Plasmodium*
*P.f.*	*Plasmodium falciparum*
*P.v.*	*Plasmodium vivax*
*P.o.*	*Plasmodium ovale*
*P.m.*	*Plasmodium malariae*
YOLO	You Only Look Once
PAM	Parasite Attention Module
PBS	Peripheral Blood Smear
CAD	Computer-Aided Diagnosis
CNN	Convolutional Neural Network
CBAM	Convolutional Block Attention Module
NAM	Normalized Attention Module
AP	Average Precision
